# Mild Adverse Events of Sputnik V Vaccine in Russia: Social Media Content Analysis of Telegram via Deep Learning

**DOI:** 10.2196/30529

**Published:** 2021-11-29

**Authors:** Andrzej Jarynowski, Alexander Semenov, Mikołaj Kamiński, Vitaly Belik

**Affiliations:** 1 System Modeling Group Institute for Veterinary Epidemiology and Biostatistics Freie Universität Berlin Berlin Germany; 2 Interdisciplinary Research Institute Wrocław/Głogów Poland; 3 Herbert Wertheim College of Engineering University of Florida Gainesville, FL United States; 4 Center for Econometrics and Business Analytics St. Petersburg State University Saint Petersburg Russian Federation

**Keywords:** adverse events, Sputnik V, Gam-COVID-Vac, social media, Telegram, COVID-19, Sars-CoV-2, deep learning, vaccine safety

## Abstract

**Background:**

There is a limited amount of data on the safety profile of the COVID-19 vector vaccine Gam-COVID-Vac (Sputnik V). Previous infodemiology studies showed that social media discourse could be analyzed to assess the most concerning adverse events (AE) caused by drugs.

**Objective:**

We aimed to investigate mild AEs of Sputnik V based on a participatory trial conducted on Telegram in the Russian language. We compared AEs extracted from Telegram with other limited databases on Sputnik V and other COVID-19 vaccines. We explored symptom co-occurrence patterns and determined how counts of administered doses, age, gender, and sequence of shots could confound the reporting of AEs.

**Methods:**

We collected a unique dataset consisting of 11,515 self-reported Sputnik V vaccine AEs posted on the Telegram group, and we utilized natural language processing methods to extract AEs. Specifically, we performed multilabel classifications using the deep neural language model Bidirectional Encoder Representations from Transformers (BERT) “DeepPavlov,” which was pretrained on a Russian language corpus and applied to the Telegram messages. The resulting area under the curve score was 0.991. We chose symptom classes that represented the following AEs: fever, pain, chills, fatigue, nausea/vomiting, headache, insomnia, lymph node enlargement, erythema, pruritus, swelling, and diarrhea.

**Results:**

Telegram users complained mostly about pain (5461/11,515, 47.43%), fever (5363/11,515, 46.57%), fatigue (3862/11,515, 33.54%), and headache (2855/11,515, 24.79%). Women reported more AEs than men (1.2-fold, *P*<.001). In addition, there were more AEs from the first dose than from the second dose (1.1-fold, *P*<.001), and the number of AEs decreased with age (*β*=.05 per year, *P*<.001). The results also showed that Sputnik V AEs were more similar to other vector vaccines (132 units) than with messenger RNA vaccines (241 units) according to the average Euclidean distance between the vectors of AE frequencies. Elderly Telegram users reported significantly more (5.6-fold on average) systemic AEs than their peers, according to the results of the phase 3 clinical trials published in *The Lancet*. However, the AEs reported in Telegram posts were consistent (Pearson correlation *r*=0.94, *P*=.02) with those reported in the Argentinian postmarketing AE registry.

**Conclusions:**

After the Sputnik V vaccination, Russian Telegram users reported mostly pain, fever, and fatigue. The Sputnik V AE profile was comparable with other vector COVID-19 vaccines. Discussion on social media could provide meaningful information about the AE profile of novel vaccines.

## Introduction

The current COVID-19 pandemic is one of the most critical global health problems. The main strategies for its mitigation involve both nonpharmaceutical interventions (eg, testing and contract tracing) and up-to-date anti-COVID-19 treatments. However, the most promising intervention has been vaccines that have effectively prevented severe COVID-19 outcomes. In addition to novel messenger RNA (mRNA) vaccines, vector vaccines have been developed. One of the first was Gam-COVID-Vac (Sputnik V), which is a viral, 2-dose, vector vaccine based on 2 human adenoviruses. Each dose contains a different vector: rAd26 and rAd5. This vaccine was developed by the Gamaleya Research Institute of Epidemiology and Microbiology. Sputnik V contains a gene that encodes SARS-CoV-2’s spike (S) protein [1]. As of the time of this manuscript submission, 2 reports of clinical trials had been published. In the first study, phases 1/2 involved a total of 76 participant, who were included in the safety analysis [2]. The report on the phase 3 trial included detailed descriptions of serious and rare adverse events (AE) as well as mild AEs described in individuals [3] older than 60 years. The overall frequency of AEs was mentioned without complete characteristics of the safety profile, such as the co-occurrence of AEs. Mild AEs are common among all vaccines. Extensive fact sheets on AEs, as well as possible adverse reactions, were provided for vaccines trialed under the US Food and Drug Administration (FDA), UK Medicines and Healthcare products Regulatory Agency (MHRA), or EU European Medicines Agency (EMA), which was not the case with Sputnik V. As of April 17, 2021 (the end of the period for collecting data in our sample), 15,700,803 single doses of COVID-19 immunization had been administered in Russia [4]. The vast majority were of the Sputnik family (>95%), and the share of other vaccines was minimal (4.7% for EpiVacCorona and 0.1% for CoviVac) [5]. Moreover, the Russian Federation had signed contracts with dozens of countries to deliver 1.4 billion doses at less than €7 (US $8.13) per dose for international buyers [6]. Therefore, there is an emerging need to update the information on Sputnik V’s safety profile using postmarketing surveillance. Because a registry of AEs after vaccination with Sputnik V is difficult to access, social media discourse may be an alternate source of information on AEs. The Sputnik vaccine gave rise to dubious situations in not only its safety profile but also other aspects [7].

An increasing number of studies has analyzed English-language social media in the context of vaccinations [8] or vaccine-prevented infectious disease [9]. However, only a few similar studies on Russian social media have been published [10,11]. Accounts of adverse reactions to drugs have been widely extracted from social media [12] in the context of mining consumer reviews on the internet [13]. To date, most of these studies processed data collected from Twitter [14-23]. Although social media platforms such as Twitter and Facebook are used in Russia, Telegram Messenger is ranked second in the Russian App Store, having 27 million active users in Russia [24]. Developed in Russia, this platform is much more popular than alternatives such as Twitter [25].

Most previous studies on social media vaccine discourse have focused on the personal beliefs of users. For example, Wang et al [26] developed a framework to detect vaccine AEs mentioned by Twitter users. However, to date, no study has analyzed social media discourse on nonsevere AEs in response to COVID-19 vaccines. In this study, we collected social media (a Telegram group in the Russian language) data to bridge the gap in information on the most prevalent AEs involving Sputnik V. We focused on the most common AEs and established which were the most prevalent, their co-occurrence, and their associations with users’ characteristics [27]. Finally, we compared the AE profile of Sputnik V with those of other approved COVID-19 vaccines.

## Methods

The dataset analyzed in our study was collected retrospectively from the Telegram group, “Sputnik_results“ [28]. The data contained no personal information, and the analysis was performed according to the Terms of Service of the platform [29]. Our analysis was completely anonymous and performed in aggregated form. No possible harm to Telegram users was identified. Therefore, the study did not require ethical committee approval.

### Data Description

Originally, Telegram aimed to provide secure communication (which is very important for post-Soviet societies [30]), but later, functionality was expanded; it added support for public channels, groups, video calls, and many other features [29]. Telegram groups may be public or private. If a group is public, it may be accessed via the Telegram search engine, and every user may read all its content. A main priority claimed by Telegram is security; users’ data are not disclosed, and only the user's screen name and picture are shown to the public. The largest Telegram channels have millions of subscribers.

The description of the “Sputnik_results” [28] public group states that its main aim is to collect information on AEs regarding the Sputnik V vaccine. Telegram users may post a description of their symptoms. Moderators of the group oversee the messages and verify that they contain only descriptions of AEs; otherwise, the message is deleted. An example message is as follows: “М, 33 года. V1 24.01.21 через 12 часов темп 39, боль в руке (все плечо целиком, мышцы), заложенность носа, диарея. На след день темп 38, боль в руке, заложенность носа. На третий день слабость, температура в норме” (translation: M, 33 years old. V1 24.01.21 after 12 hours, temp. 39, pain in the arm (the entire shoulder, muscles), nasal congestion, diarrhea. The next day, temp. 38, pain in the arm, nasal congestion. On the third day, weakness, temperature is normal).

In this study, we collected all messages from the “Sputnik_Results” group using Python Telegram Client telethon [31]. We saved only text messages that were posted in the group; users’ personal details were not extracted. In total, we collected 18,833 messages. After filtering messages that contained only pictures, 11,515 messages remained. The first message was sent on December 9, 2020, and the most recent message was sent on April 17, 2021. The dataset contained 25,660 unique lowercase words.

### Adverse Event Classification

The gold standard used to identify adverse reactions is the MedDRA System Organ Class, which is applied in the European Union (EudraVigilance [32]), the United States (Vaccine Adverse Event Reporting System [VAERS] [33]), and the United Kingdom (MHRA Yellow Card scheme [34]). However, the system uses a specialized medical vocabulary. In our study, because users of social media communicated in colloquial language [12], we chose a simplified FDA classification system [35-37] that was subdivided into 2 groups: local reactions (ie, redness, swelling, and pain at the injection site) and systemic reactions (ie, fever, fatigue, headache, chills, nausea/vomiting, diarrhea, new or worsening muscle pain, and new or worsening joint pain). Moreover, muscle pain, joint pain, and pain at the injection site were categorized as a single class. However, we added the classes of pruritus, enlarged lymph nodes, and insomnia, which are common adverse reactions to anti-COVID-19 vaccines [38,39]. Insomnia was chosen due to its high frequency by simple keyword analysis on a sample of material from Telegram. The final list of 12 classes of symptoms of mild AEs, which were based on subjective experiences of a potential health issue, is provided in the Results section.

### Labeling

We utilized the LabelStudio data labeling tool [40] to label the dataset. We randomly sampled 1000 messages in the dataset, which were labeled by 3 raters who were native Russian speakers. The raters labeled each occurrence of an AE in the messages, thus making the dataset suitable for named entity recognition tasks. Because of such labeling and the existence of different descriptions of the same AEs in multiple sentences, we augmented the dataset by splitting each message into sentences. The resulting dataset contained 4579 entities.

### Model Architecture

Each message in our dataset could have included multiple AEs. We therefore adopted a multilabel text classification scheme. A formal definition of multilabel classification is as follows: Consider a dataset




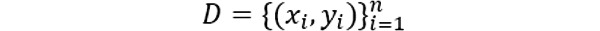




where *x_i_* ε *X* is the *i*-th observed variable for the dataset of cardinality *n*, *y_i_* ε *Y* is the corresponding set of labels for the *i*-th element. Our goal was to learn a mapping *ŷ_j_* = *f(x_j_*,θ), where *ŷ_j_* is the set of predicted classes and *θ* is a vector of parameters. To find the vector of optimal parameters *θ*, we needed to minimize the loss function *L*(*y*,*ŷ*) between the actual and predicted classes. Multiple machine learning methods may be applied to support multilabel classification. In the case of artificial neural networks (ANNs), the activation function of the last layer of the ANN is set to be a sigmoid:




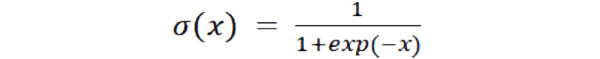




and binary cross-entropy loss is used. In this case, ANN will map the probability of each class to a value between 0 and 1, and each data item could be mapped to multiple classes.

Because of the recent success of ANNs, specifically transformers, in text analysis tasks, we adopted a deep Bidirectional Encoder Representations from Transformers (BERT) architecture to perform our multilabel classification task [41]. We utilized a pretrained BERT model for the Russian language DeepPavlov [42]. We tuned the last layer of the model, which consisted of 12 sigmoid neurons. As a baseline, we used a standard long short-term memory (LSTM) ANN, which consisted of embedding as the first layer and 1 LSTM layer (100 cells), dropout (*P*=.20), and a subsequent multilabel dense layer with sigmoid as the activation function.

### Model Evaluation

We trained the BERT and LSTM models using a stratified k-fold validation scheme where *k*=5. Because the classes were imbalanced, we utilized an up-sampling strategy; that is, underrepresented classes were up-sampled in the training dataset. The testing set distribution was not modified. Table 1 displays the evaluation results. Precision and recall were calculated for both micro- and macro-averaged aggregations [43]. As shown in Table 1, precision and F1 scores were reported for thresholds equal to 0.5. We utilized a computer with a Tesla T4 GPU to train the models. Table 1 shows that BERT outperformed the LSTM model by a large margin. We therefore chose the BERT model and trained it on 95% of the data; in this case, it returned a micro-averaged accuracy of 0.94 and an area under the receiver operating characteristic (ROC) curve (AUC) score of 0.991.

Regarding gender, age, and dose number (if available), we used counts of corresponding abbreviations and regular expression matching because the administrators of the group had provided detailed instructions for the reporting of this information.

**Table 1:** Bidirectional Encoder Representations from Transformers (BERT) and long short-term memory (LSTM) model evaluation results.

Model	Micro-averaged aggregations				Macro-averaged aggregations	
	AUC^a^, mean (SD)	Precision, mean (SD)	F1, mean (SD)	Precision, mean (SD)		F1, mean (SD)
LSTM	0.969 (0.002)	0.866 (0.024)	0.769 (0.033)	0.514 (0.048)		0.431 (0.042)
BERT	0.991 (0.002)	0.915 (0.016)	0.920 (0.002)	0.863 (0.025)		0.858 (0.006)

^a^AUC: area under the curve.

### Analysis of AE

To evaluate the time relationship between the number of reports and vaccination volume, a univariate linear regression coefficient was calculated. Because the number of reports (*P*<.001) and vaccination volume (*P*<.001) failed to be normally distributed based on the Shapiro-Wilk test, a Spearman correlation was calculated. Because the number of AEs failed to be normally distributed based on the Shapiro-Wilk test (*P*<.001), the difference between the 2 groups was analyzed with a Mann-Whitney U test. To compare frequencies of AEs between 2 samples of AEs, a Fisher test was applied. To compare the frequencies of 2 vectors of AEs, the normality was checked with the Shapiro-Wilk test, and Pearson correlations could be calculated (*P*=.10 and *P*=.07, respectively, comparing Telegram with the Argentinian Registry; *P*=.13 and *P*=.34, respectively, comparing Telegram with the Moscow trial). Community detection was conducted to evaluate the internal structure (co-occurrence) of AEs in the network representation.

## Results

Reactogenicity assessment based on opt-in civic surveillance was performed to obtain results of clinical importance (similar to endpoints in trials).

### Temporal Dynamics

**Figure 1:**
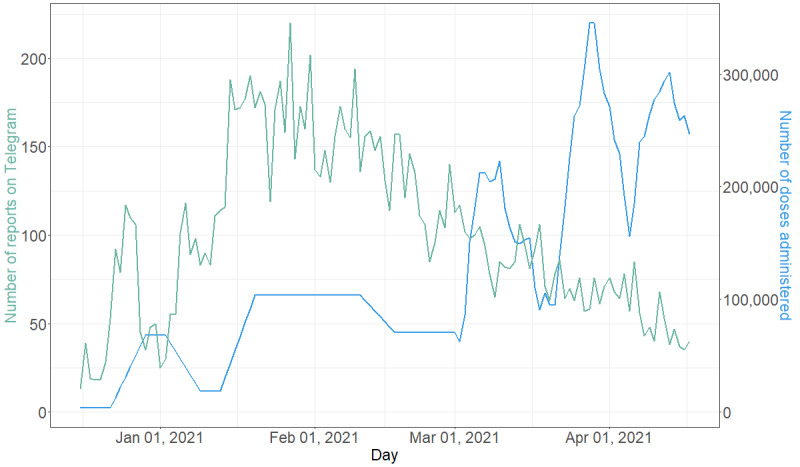
Daily counts of reports of adverse events (AE) and doses administered in Russia (data according to Our World in Data [4,44]).

The peak in the volume of self-reports corresponded with the time at which vaccinations were sped up (Figure 1). Moreover, after 3 months of vaccinations (the end of February 2021), the popularity of self-reporting started to decrease despite the increasing vaccination roll-out. However, the Spearman correlation coefficient between the volume of self-reports and doses administered from December 9, 2020 until February 28, 2021 was very high (*r*=0.75, *P*<.001), and the subsequent count of administered doses increased, while reports on AEs decreased (Figure 1).

### Revealed AE Frequencies (BERT Classes)

Our analysis revealed that fever and generalized pain were the most commonly reported AEs (Table 2). Injection site irritations (local reactions) were an order of magnitude less likely to be reported than fever and pain (systemic reaction). Gastric symptoms (especially diarrhea, with a frequency of 0.6% per report) were less likely to be reported than the average prevalence among the general population (1%-5% for diarrhea [45,46]).

**Table 2:** Frequencies of mild adverse events extracted from the Telegram group (n=11,515).

Adverse events			n (%)
**Systemic**			
	Fever	5461 (47.43)	
	Pain	5363 (46.57)	
	Fatigue	3862 (33.54)	
	Headache	2855 (24.79)	
	Chills	2651 (23.02)	
	Insomnia	600 (5.21)	
	Lymph node enlargement	186 (1.62)	
**Local**			
	Erythema/redness	319 (2.77)	
	Swelling	206 (1.79)	
	Pruritis	199 (1.73)	
**Gastric**			
	Nausea/vomiting	351 (3.05)	
	Diarrhea	66 (0.57)	

### Variations Across Age, Gender, and Dose

Gender was reported by 3992 women and 2762 men. On average, women reported 2.5 AEs (*σ*=1.79; Q1=1; Q2=2; Q3=4), and men reported 2.1 AEs (*σ*=1.64; Q1=1; Q2=2; Q3=3). Women reported statistically significantly more AEs (*P*<.001) according to the results of a Mann-Whitney U test (Table 3).

**Figure 2:**
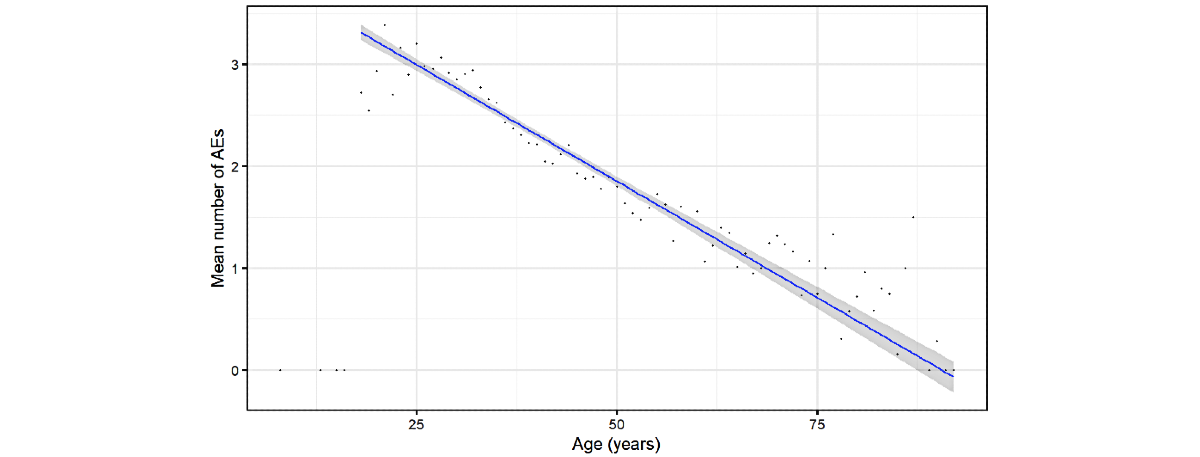
Scatterplot of the number of adverse events (AEs) reported by user vs. age. Dots indicate the mean number of AEs for a given age, while the blue line indicates the linear regression trend and shadowed area indicate its CIs.

Age was provided by 6754 users. A linear regression analysis was performed for those who reported being at least 18 years old (minimal age of Russian registration [1]). We found a clear and significant linear relationship (*β*=.0457, SE=.0014), showing that with every year of life, users reported .0457 fewer AEs (Figure 2). In univariate regression analysis, *β* is an estimated coefficient with a given SE. Mild AEs among the elderly are known to be less frequently observed for most anti-COVID-19 vaccines [35-37,47].

AEs in response to other anti-COVID-19 vaccines have been found to depend on whether the vaccination was the first or the second dose (if applicable). For instance, AEs in response to mRNA vaccines have tended to be stronger with the second dose [36,37,47]. In contrast, AEs in response to vector vaccines have tended to be milder with the second dose [48,49]. Regarding the Sputnik V vaccine, this difference might be because a different vector is used in each dose, which might lead to different reactions. Among the self-reports, 4174 described AEs after the first dose, 1251 described AEs after the second dose, and 3049 described AEs after both doses. It is also possible that the users did not receive the second dose because of contraindications or just lost interest in reporting.

Here, we considered only reports that discussed the first and second doses separately. On average, there were 2.2 (*σ*=1.80; Q1=0; Q2=2; Q3=4) AEs for the first dose and 1.9 (*σ*=1.69; Q1=0; Q2=2; Q3=3) AEs for the second dose. According to the results of the Mann-Whitney U test, there were statistically significantly more AEs after the first dose (*P*<.001; Table 3).

**Table 3:** Comparisons of the mean numbers of adverse events (AEs) by gender and by dose using Mann-Whitney U tests.

Variable			Number of AEs, mean		OR^a^		*P* value
**Gender**							
	Male	2.1		1.20		<.001	
	Female	2.5					
**Dose**							
	First	2.2		1.13		<.001	
	Second	1.9					

^a^OR: odds ratio.

### Co-occurrence of AEs

**Figure 3:**
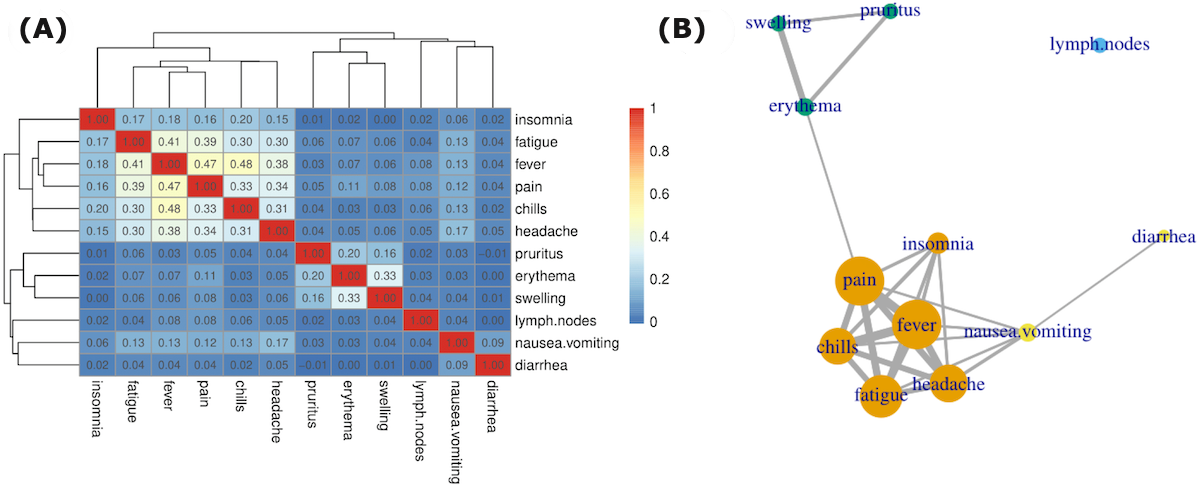
Co-occurrence of adverse events (AEs), shown as (A) hierarchical clustering based on the correlation matrix of AE symptoms and (B) the corresponding network of AE symptoms with different communities denoted by color code.

To quantify the co-occurrence of symptoms, we calculated Spearman rank correlation coefficients between each pair of classified symptoms. We observed systemic, local, and gastric clusters (Figure 3). We also provided a network representation in which vertex size represents symptom prevalence and edge width represents co-occurrence as measured by the correlation coefficient. Only edges with a correlation coefficient above 0.09 are shown (Figure 3). An unsupervised weighted Louvain algorithm [50] for community detection was used for this purpose, and the vertices were colored the same if they belonged to the same community, which revealed a meaningful structure in which orange denoted systemic, green denoted local, and yellow denoted gastric communities of symptoms.

### Telegram Versus Other Trials or Registries of Sputnik V

We compared our results with 2 available datasets of AEs in response to the Sputnik V vaccine. The first one was collected in Moscow. The second one was collected in Argentina.

#### Moscow Clinical Trial

Mild AEs in 1029 patients older than 60 years in the phase 3 clinical trial [3,51] in Moscow were compared with 690 self-reports by Telegram users older than 60 years (Table 4). Because there were inconsistencies in various definitions of AEs, a simplified classification was provided, and only headache and diarrhea comprised similar symptoms (at least *sensu lato*).

We performed the following calculations to compare both datasets. To obtain *fever* according to our definition, we summed the results for pyrexia, fever sensation, and elevated body temperature from the clinical trial. Similarly, to obtain *pain,* we summed the results for myalgia, arthralgia, and local reaction. To obtain *fatigue,* we summed the results for asthenia and malaise. To obtain *nausea,* we summed the results for nausea and dyspepsia. For *erythema,* we chose the results for contact dermatitis.

In all systemic reactions, Telegram users reported AEs significantly more often than measured in the clinical trial (Table 4). In contrast, diarrhea was less likely to be reported than measured in the clinical trial.

**Table 4:** Comparisons of adverse events with the Sputnik vaccine between the Telegram and Moscow clinical trial [3] datasets (*r*=0.69, *P*=.09).

Adverse event	Moscow clinical trial, n (%)	Telegram, n (%)	OR^a^	*P* value^b^
Pain	67 (6.70)	177 (25.65)	3.82	<.001
Headache	30 (2.92)	89 (12.90)	4.42	<.001
Fatigue	31 (3.01)	141 (20.43)	6.78	<.001
Fever	32 (3.11)	163 (23.62)	7.59	<.001
Nausea	12 (1.17)	9 (1.30)	1.12	.83
Erythema	39 (3.79)	15 (2.17)	0.57	.09
Diarrhea	8 (0.78)	3 (0.43)	0.56	.54

^a^OR: odds ratio for the Moscow clinical trial.

^b^Fisher test results for the comparison between samples.

#### Argentinian Postregistration AE Registry

Another available dataset on AEs in response to Sputnik V was compiled from the Argentinian registry of passive AE monitoring (Table 5). This registry contains 23,804 events of all kinds of AEs (mild AEs: 22,971/23,804, 96.5%) from 2,541,362 doses administered. To compare, we chose 7797 Telegram posts that reported at least one AE, and we adjusted new disjoint subsets of symptoms according to the Argentinian methodology [44].

We categorized gastric as the frequency of the logical function nausea OR diarrhea. We categorized site irritation as the frequency of the logical function pruritus OR erythema OR swelling. We categorized fever_pain as the frequency of the logical function fever AND (pain OR headache). We categorized fatigue_pain as the frequency of the logical function fatigue AND (pain OR headache). We categorized only_fever as the frequency of the logical function fever AND ˜(pain OR headache OR fatigue); ˜ denotes logical negation.

The comparison showed that the statistics, despite the significant differences shown in Table 5, were similar in magnitude and highly correlated (*r*=0.94). The comparison of the Telegram reports (a selected sample with at least one AE constructed by multilabel classification) with the Argentinian registry (multiclass classification [44]) was conducted by the aforementioned mapping. The results of the comparison must be interpreted with caution.

**Table 5:** Comparisons of adverse events with the Sputnik vaccine between the Telegram and Argentinian safety monitoring [44] datasets (*r*=0.94, *P*=.02).

Adverse event	Argentinian registry, n (%)	Telegram, n (%)	OR^a^	*P* value^b^
fever_pain	8210 (33.25)	4142 (54.70)	1.66	<.001
fatigue_pain	9407 (38.10)	2998 (39.67)	1.05	.05
gastric	1447 (5.98)	395 (5.14)	0.90	.07
site irritation	2306 (9.34)	558 (7.31)	0.80	<.001
only_fever	2065 (8.34)	697 (9.53)	1.11	.02

^a^OR: odds ratio for the Argentinian registry.

^b^Fisher test results for the comparison between samples.

### Comparison With Other Vaccines

**Figure 4:**
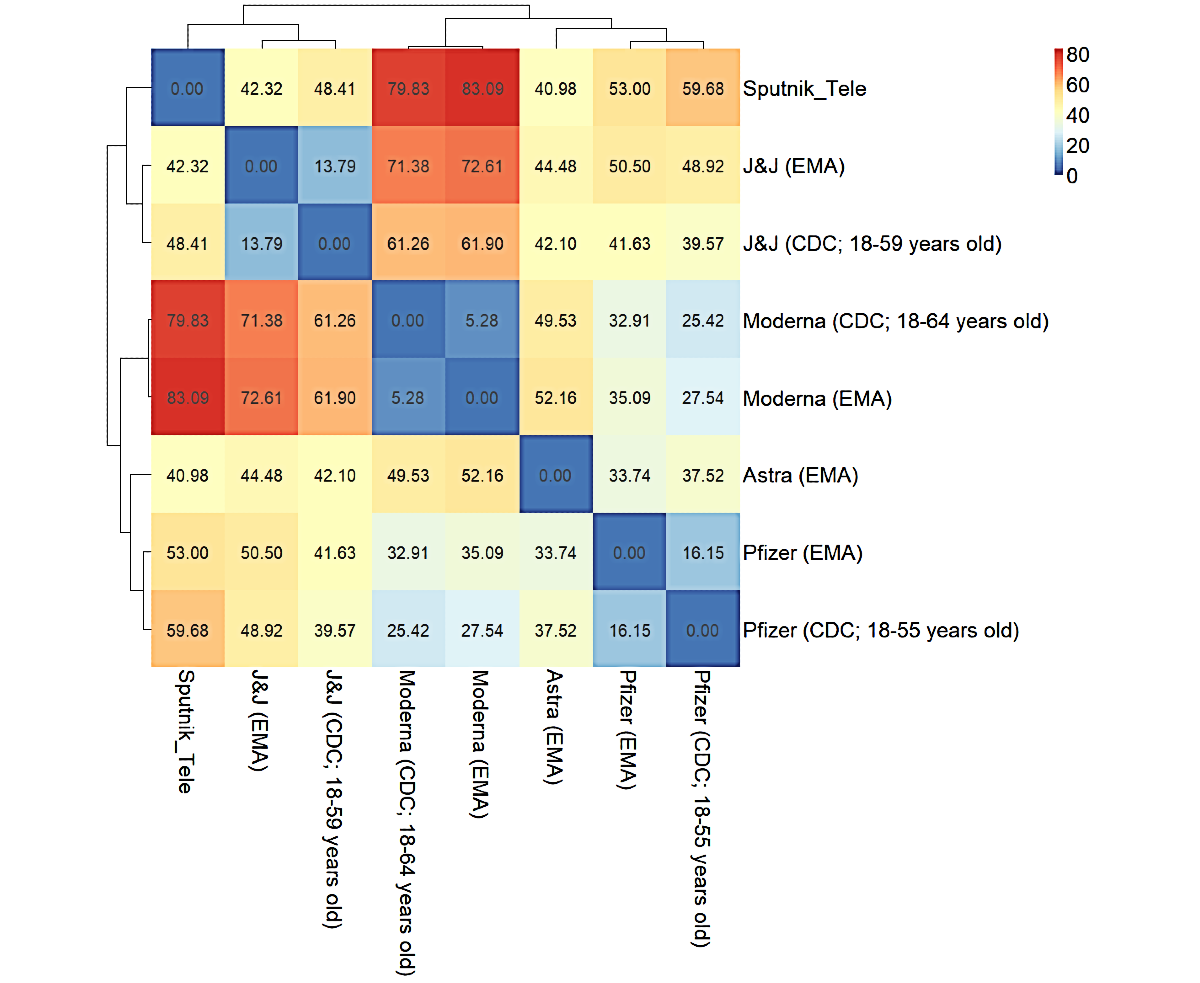
Hierarchical matrix of adverse event (AE) similarity of various vaccines and reporting systems (Euclidean distance) of vaccinations investigated in the present study. Astra: AstraZeneca; CDC: Centers for Disease Control and Prevention; EMA: European Medicines Agency; FDA: Food and Drug Administration; J&J: Johnson & Johnson.

Regarding vaccines registered by the EMA and FDA, lists of the frequencies of the most common adverse events are accessible; however, they vary across regulatory bodies. Thus, we chose a subset of symptoms for frequencies that were reasonably comparable (pain, headache, fatigue, fever, chills, and nausea). We built a distance (Euclidean) matrix of AEs based on clinical trial registries (EMA [48,52-54], FDA [35-37]) and from the Telegram group (Table 6). From the FDA dataset, for 2-dose vaccines, the dose with higher reactogenicity was selected. In clinical trials, pain is usually considered as pain at the injection site. Fever was the sum of pyrexia and fever in the EMA database. EMA used the injection site tenderness/irritation category. However, regarding redness/erythema, the FDA classified swelling and pruritus separately. Thus, erythema was not included. Sputnik V is a vector vaccine, as are those from AstraZeneca and Johnson & Johnson. The results showed that Telegram Sputnik V AEs were clustered with other vector vaccines, which was possibly due to similar safety profiles (Figure 4).

It is important to note that the Telegram users also submitted reports without any AEs at all. Thus, our surveillance system included a sentinel property of samples in contrast to VAERS (North America), EudraVigilance (European Union), and the Argentinian registry [44], which gather reports only if there is any AE to be reported.

**Table 6:** Adverse events in response to Sputnik V (Telegram) and other vaccines (European Medicines Agency [EMA] and Centers for Disease Control and Prevention [CDC]/Food and Drug Administration [FDA]).

Vaccine	Pain, n (%)	Headache, n (%)	Fatigue, n (%)	Fever, n (%)	Chills, n (%)	Nausea, n (%)
AstraZeneca (EMA)	–^a^ (54.20)	– (52.60)	– (53.10)	– (41.50)	– (31.90)	– (21.80)
Johnson & Johnson (EMA)	– (48.60)	– (38.90)	– (38.20)	– (14.00)	– (5.00)	– (14.20)
Johnson & Johnson (CDC; 18-59 years old)	1193 (59.80)	905 (44.40)	891 (43.80)	261 (12.80)	– (5.00)	315 (15.50)
Pfizer (EMA)	– (80.00)	– (50.00)	– (60.00)	– (30.00)	– (30.00)	– (5.00)
Pfizer (CDC; 18-54 years old)	1632 (77.80)	1085 (51.70)	1247 (59.40)	331 (15.80)	737 (35.10)	– (10.00)
Sputnik (Telegram)	5363 (46.57)	2855 (24.80)	3862 (33.54)	5461 (47.43)	2651 (23.02)	351 (3.00)
Moderna (CDC; 18-64 years old)	9335 (90.10)	6500 (62.80)	7002 (67.60)	1806 (17.40)	5001 (48.30)	2209 (21.30)
Moderna (EMA)	– (92.00)	– (64.70)	– (70.00)	– (15.50)	– (45.40)	– (23.00)

^a^Not reported.

## Discussion

### Principal Findings

According to clinical trials [3] and official registries [44], only partial information could be retrieved on the Sputnik V safety profile. Previously, multiple researchers have raised concerns about the safety of the Sputnik V vaccine [6,55,56]. Our study aimed to increase transparency regarding the safety of Sputnik V [57], because drug regulatory agencies such as in Brazil were delaying Sputnik V emergency registration: “Anvisa was unable to validate the methodology Russian studies used (...) to track and describe adverse events following vaccination” [58]. In this study, we showed that community-based surveillance via social media can provide meaningful information that could be useful, and this phenomenon should be carefully investigated. The frequencies of AEs extracted from Telegram samples in which at least one AE was reported were in line with other safety surveillance.

Mild, nonsevere AEs have usually been ignored by medical communities because they are common to all vaccines. Antivax movements have emphasized severe AEs, which have been widely discussed in social media [59] in the wider context of vaccine safety [60,61]. In the discourse on COVID-19 vaccines, the main issues were that they were developed quickly and they could compromise safety. Those issues included the fear that vaccines would alter human DNA, cause allergic reactions to vaccine ingredients, result in sudden deaths due to frailty syndrome, or cause infertility [62,63]. Wide anti-COVID-19 immunization programs promulgated a discourse in which risk (eg, the discomfort of common, but mild, AEs as well as rare, but serious AEs) and benefits (eg, efficacy in protecting from the disease) were described as “tradeoffs” of being vaccinated. Mild AEs have become an important issue for many people; moreover, they have the economic component of the potential need for sick leave. This discourse led to the formation of a public Telegram group, where users were asked to report AEs.

In this study, we demonstrated that, in the first phase of the vaccination roll-out, the AE reports were correlated (*r*=0.7) with vaccination volume (Figure 1). However, Telegram users tended to lose interest after a few months. It is possible that because of the prioritization of vaccine delivery, which began with public and military servants, scientists, teachers, and medical staff, these “early adopters” were more likely to post on social media and be actively involved in reporting AEs. Subsequently, users in the general population were vaccinated, and they were less involved in reporting on the Telegram platform (Figure 1). Thus, interest in COVID-19, Sputnik V, and its AEs was influenced by social context and media to much extent [64].

The results of this study showed that the number of reported AEs decreased linearly according to age (*β*=.05 AE per year; Figure 2). This result was dependent on biology, which was confirmed in previous clinical trials [36,37,52] and postmarketing observations [47] of other anti-COVID-19 vaccines. Telegram users older than 60 years reported significantly more systemic AEs compared with their peers in clinical trials, who tested negative for or had recovered from COVID-19 [51] (Table 4). On one hand, it is possible that people previously infected with COVID-19 were more likely to report AEs after receiving other vaccines [47]. On the other hand, self-reporting bias could be an important factor in explaining the difference between the “Moscow” clinical trial and the Telegram reports.

The safety profile of the Sputnik V vaccine includes mild AEs that are more similar to vector vaccines than to mRNA anti-COVID-19 vaccines, which was quantified by the Euclidean distance between AE frequencies (Figure 4). The Sputnik V safety profile also showed a high fever-to-fatigue ratio (Table 6) and a stronger reaction to the first dose than to the second one (Table 3), which was also analyzed in a retrospective observational study in San Marino [65].

Women reported more AEs than men (1.2-fold, *P*<.001; Mann-Whitney U test). This phenomenon is well recorded in other anti-COVID-19 vaccine registries [47,66] and has even been noticed among Argentinian medical staff [67], which could indicate sex-dependent vaccine reactivity. However, this result needs to be understood with caution. The Centers for Disease Control and Prevention has warned that gender bias in reporting could be more important than possible biological mechanisms [35]. The likelihood of disclosing personal information (even anonymously) is known to vary, such as according to gender [68] and social class [69]. A potential reason is that women are more likely to be interested in health, write about health on the internet, and disclose their information [68].

On Telegram, self-reports are most likely to underestimate gastric symptoms (eg, diarrhea at 0.6%). These symptoms could be a taboo effect [70], such as a response to public speaking anxiety. Alternatively, it could be easily ignored because of its high prevalence, or it could be eliminated using an over-the-counter medicine such as loperamide [45,46]. Insomnia was detected so often that it suggests an epidemiological link with the vaccine, which needs further investigation. Local AEs, such as injection site irritation, have rarely been reported. Underlying conditions of erythema/redness, which is usually one of the most common AEs in response to all injected substances including vaccines, are probably overlooked due to low subjective discomfort and lack of physical investigation by a doctor. The findings showed that their actual prevalence was probably underreported.

Our study has several limitations. First, we analyzed participatory and community-based surveillance among Russian Telegram users. Therefore, the results may be specific to the Russian population in a given stage of the pandemic and therefore should not be extrapolated to other contexts. Second, Telegram users may overlook less troublesome side effects, and the social context could influence decisions on taking part in discussions and being selective in reporting AEs [68,69]. For example, local or gastric AEs could be underreported. Third, the classifications developed in this study should not be strictly applied to other contexts. For example, pain at the injection site and pain in other parts of the body were not differentiated. Observed correlations and odds ratios do not imply causation. Fourth, we did not assess the authenticity and credibility of posts [15]; thus, incorrect information could be included in the data. Finally, because our infodemiology study focused on community research initiatives (independent and nonprofit projects, with already known strengths and weaknesses from the history of medicine [71]), our observations cannot replace real-world studies [55-57]. The symptoms reported by social media users only partially reflect their prevalence in the real world [72]. Therefore, the frequencies of symptoms should not be interpreted without considering the contexts and proportions of other symptoms (ie, fever-to-fatigue ratio), phase of the epidemic, and vaccination roll-out (ie, the number of doses administered daily and the population that is vaccinated), as willingness to report AEs satisfies typical product life-cycle temporal characteristics [73,74]).

### Conclusion

After the Sputnik V vaccination, Russian Telegram users reported mostly pain, fever, and fatigue. The Sputnik V mild AE profile was comparable with other vector COVID-19 vaccines. Discussions on social media could provide meaningful information about the AE profile of novel vaccines. Further research on severe AEs reported on social media and their credibility is needed.

## Abbreviations

AE: adverse event
ANN: artificial neural network
AUC: area under the curve
BERT: Bidirectional Encoder Representations from Transformers
EMA: European Medicines Agency
FDA: Food and Drug Administration
LSTM: long short-term memory
MHRA: Medicines and Healthcare products Regulatory Agency
ROC: receiver operating characteristic
VAERS: Vaccine Adverse Event Reporting System

## References

[1] Gam-COVID-Vac: Combined vector vaccine for the prevention of coronavirus infection (in Russian). State Register of Medicines.

[2] Logunov DY, Dolzhikova IV, Zubkova OV, Tukhvatulin Ai, Shcheblyakov DV, Dzharullaeva AS, Grousova DM, Erokhova AS, Kovyrshina AV, Botikov AG, Izhaeva FM, Popova O, Ozharovskaya TA, Esmagambetov IB, Favorskaya IA, Zrelkin DI, Voronina DV, Shcherbinin DN, Semikhin AS, Simakova YV, Tokarskaya EA, Lubenets NL, Egorova DA, Shmarov MM, Nikitenko NA, Morozova LF, Smolyarchuk EA, Kryukov EV, Babira VF, Borisevich SV, Naroditsky BS, Gintsburg AL (2020). Safety and immunogenicity of an rAd26 and rAd5 vector-based heterologous prime-boost COVID-19 vaccine in two formulations: two open, non-randomised phase 1/2 studies from Russia. The Lancet.

[3] Logunov DY, Dolzhikova IV, Shcheblyakov DV, Tukhvatulin AI, Zubkova OV, Dzharullaeva AS, Kovyrshina AV, Lubenets NL, Grousova DM, Erokhova AS, Botikov AG, Izhaeva FM, Popova O, Ozharovskaya TA, Esmagambetov IB, Favorskaya IA, Zrelkin DI, Voronina DV, Shcherbinin DN, Semikhin AS, Simakova YV, Tokarskaya EA, Egorova DA, Shmarov MM, Nikitenko NA, Gushchin VA, Smolyarchuk EA, Zyryanov SK, Borisevich SV, Naroditsky BS, Gintsburg AL (2021). Safety and efficacy of an rAd26 and rAd5 vector-based heterologous prime-boost COVID-19 vaccine: an interim analysis of a randomised controlled phase 3 trial in Russia. The Lancet.

[4] Devlin K, Connaughton A (2020). Most Approve of National Response to COVID-19 in 14 Advanced Economies. Pew Research Center.

[5] (2021). The Ministry of Industry and Trade told about the number of vaccines released into circulation in Russia. TASS.

[6] Baraniuk C (2021). Covid-19: What do we know about Sputnik V and other Russian vaccines?. BMJ.

[7] Bucci EM, Berkhof J, Gillibert A, Gopalakrishna G, Calogero RA, Bouter LM, Andreev K, Naudet F, Vlassov V (2021). Data discrepancies and substandard reporting of interim data of Sputnik V phase 3 trial. Lancet.

[8] Karafillakis E, Martin S, Simas C, Olsson K, Takacs J, Dada S, Larson HJ (2021). Methods for social media monitoring related to vaccination: Systematic scoping review. JMIR Public Health Surveill.

[9] Samaras L, García-Barriocanal E, Sicilia MA (2020). Syndromic surveillance using web data: a systematic review. Innovation in Health Informatics.

[10] Zheluk A, Gillespie JA, Quinn C (2012). Searching for truth: internet search patterns as a method of investigating online responses to a Russian illicit drug policy debate. J Med Internet Res.

[11] Sboev AG, Sboeva SG, Gryaznov AV, Evteeva AV, Rybka RB, Silin MS (2020). A neural network algorithm for extracting pharmacological information from russian-language internet reviews on drugs. J. Phys.: Conf. Ser.

[12] Dai X, Karimi S, Paris C (2017). Medication and adverse event extraction from noisy text. Proceedings of Australasian Language Technology Association Workshop.

[13] Salehan M, Kim D (2016). Predicting the performance of online consumer reviews: A sentiment mining approach to big data analytics. Decision Support Systems.

[14] Gattepaille LM, Hedfors Vidlin S, Bergvall T, Pierce CE, Ellenius J (2020). Prospective evaluation of adverse event recognition systems in Twitter: Results from the Web-RADR Project. Drug Saf.

[15] Hoang T, Liu J, Pratt N, Zheng VW, Chang KC, Roughead E, Li J (2018). Authenticity and credibility aware detection of adverse drug events from social media. Int J Med Inform.

[16] Adrover C, Bodnar T, Huang Z, Telenti A, Salathé M (2015). Identifying adverse effects of HIV drug treatment and associated sentiments using Twitter. JMIR Public Health Surveill.

[17] Zhou Z, Hultgren KE (2020). Complementing the US Food and Drug Administration Adverse Event Reporting System with adverse drug reaction reporting from social media: Comparative analysis. JMIR Public Health Surveill.

[18] Li Y, Jimeno Yepes A, Xiao C (2020). Combining social media and FDA Adverse Event Reporting System to detect adverse drug reactions. Drug Saf.

[19] Patel R, Belousov M, Jani M, Dasgupta N, Winokur C, Nenadic G, Dixon WG (2018). Frequent discussion of insomnia and weight gain with glucocorticoid therapy: An analysis of Twitter posts. NPJ Digit Med.

[20] Martínez-López De Castro N, Samartín-Ucha M, Martín-Vila A, Álvarez-Payero M, Piñeiro-Corrales G, Pego-Reigosa JM (2019). Content analysis of Twitter in relation to biological treatments for chronic inflammatory arthropathies: an exploratory study. Eur J Hosp Pharm.

[21] Smith K, Golder S, Sarker A, Loke Y, O'Connor K, Gonzalez-Hernandez G (2018). Methods to compare adverse events in Twitter to FAERS, drug information databases, and systematic reviews: Proof of concept with adalimumab. Drug Saf.

[22] Pierce CE, Bouri K, Pamer C, Proestel S, Rodriguez HW, Van Le H, Freifeld CC, Brownstein JS, Walderhaug M, Edwards IR, Dasgupta N (2017). Evaluation of Facebook and Twitter monitoring to detect safety signals for medical products: An analysis of recent FDA Safety Alerts. Drug Saf.

[23] Huesch MD (2017). Commercial online social network data and statin side-effect surveillance: A pilot observational study of aggregate mentions on Facebook. Drug Saf.

[24] (2020). Telegram audience grew by 1.2 million people in a month. TASS.

[25] Melkadze A (2021). Leading social media platforms in Russia as of 3rd quarter of 2020, by penetration rate. Statista.

[26] Wang J, Zhao L, Ye Y, Zhang Y (2018). Adverse event detection by integrating twitter data and VAERS. J Biomed Semantics.

[27] Jarynowski A (2021). Sputnik V Adverse Events risk calculator.

[28] People's reports on vaccination from Covid-19: Project V1V2.ru | reviews after vaccine vaccination against covid side effects satellite v m light. Telegram.

[29] Terms of Service. Telegram.

[30] Semenov A, Mantzaris A, Nikolaev A, Veremyev A, Veijalainen J, Pasiliao El, Boginski V (2019). Exploring social media network landscape of post-Soviet space. IEEE Access.

[31] LonamiWebs / Telethon. GitHub.

[32] EudraVigilance - European database of suspected adverse drug reaction reports. European Medicines Agency.

[33] Vaccine Adverse Event Reporting System. Department of Health and Human Services.

[34] Yellow Card. Medicines and Healthcare products Regulatory Agency.

[35] The Janssen COVID-19 Vaccine’s Local Reactions, Systemic Reactions, Adverse Events, and Serious Adverse Events. Centers for Disease Control and Prevention.

[36] The Moderna COVID-19 Vaccine’s Local Reactions, Systemic Reactions, Adverse Events, and Serious Adverse Events. Centers for Disease Control and Prevention.

[37] The Pfizer COVID-19 Vaccine’s Local Reactions, Systemic Reactions, Adverse Events, and Serious Adverse Events. Centers for Disease Control and Prevention.

[38] McDonald I, Murray SM, Reynolds CJ, Altmann DM, Boyton RJ (2021). Comparative systematic review and meta-analysis of reactogenicity, immunogenicity and efficacy of vaccines against SARS-CoV-2. NPJ Vaccines.

[39] Kaur RJ, Dutta S, Bhardwaj P, Charan J, Dhingra S, Mitra P, Singh K, Yadav D, Sharma P, Misra S (2021). Adverse events reported from COVID-19 vaccine trials: A systematic review. Indian J Clin Biochem.

[40] Label Studio. Heartex.

[41] Devlan J, Chang MW, Lee K, Toutanova K (2019). BERT: Pre-training of Deep Bidirectional Transformers for Language Understanding. Cornell University.

[42] deepmipt / DeepPavlov. GitHub.

[43] Sokolova M, Lapalme G (2009). A systematic analysis of performance measures for classification tasks. Information Processing & Management.

[44] 10th Vaccine Safety Report. Ministerio de Salud Argentina.

[45] Kamiński M, Borger M, Prymas P, Muth A, Stachowski A, Łoniewski I, Marlicz W (2020). Analysis of answers to queries among anonymous users with gastroenterological problems on an internet forum. Int J Environ Res Public Health.

[46] Panayiotou G, Karekla M, Georgiou D, Constantinou E, Paraskeva-Siamata M (2017). Psychophysiological and self-reported reactivity associated with social anxiety and public speaking fear symptoms: Effects of fear versus distress. Psychiatry Res.

[47] Menni C, Klaser K, May A, Polidori L, Capdevila J, Louca P, Sudre CH, Nguyen LH, Drew DA, Merino J, Hu C, Selvachandran S, Antonelli M, Murray B, Canas LS, Molteni E, Graham MS, Modat M, Joshi AD, Mangino M, Hammers A, Goodman AL, Chan AT, Wolf J, Steves CJ, Valdes AM, Ourselin S, Spector TD (2021). Vaccine side-effects and SARS-CoV-2 infection after vaccination in users of the COVID Symptom Study app in the UK: a prospective observational study. Lancet Infect Dis.

[48] COVID-19 Vaccine AstraZeneca - Summary of Product Characteristics. European Medicines Agency.

[49] Coronavirus vaccine - weekly summary of Yellow Card reporting. Medicines & Healthcare products Regulatory Agency.

[50] Blondel V, Guillaume J, Lambiotte R, Lefebvre E (2008). Fast unfolding of communities in large networks. J. Stat. Mech.

[51] Logunov DY, Dolzhikova IV, Tukhvatullin AI, Shcheblyakov DV (2020). Safety and efficacy of the Russian COVID-19 vaccine: more information needed - Authors' reply. Lancet.

[52] COVID-19 Vaccine Janssen-Summary of Product Characteristics. European Commission.

[53] COVID-19 mRNA Vaccine Moderna-Summary of Product Characteristics. European Commission.

[54] COVID-19 mRNA Vaccine Comirnaty-Summary of Product Characteristics. European Medicines Agency.

[55] van Tulleken C (2021). Covid-19: Sputnik vaccine rockets, thanks to Lancet boost. BMJ.

[56] Vlassov V (2021). Sputnik V and Russia’s covid-19 vaccine race. The BMJ Opinion.

[57] Logunov DY, Dolzhikova IV, Shcheblyakov DV (2021). Data discrepancies and substandard reporting of interim data of Sputnik V phase 3 trial - Authors' reply. Lancet.

[58] Brito R, Ivanova P (2021). Brazil health regulator rejects Russia's Sputnik vaccine. Reuters.

[59] Klimiuk K, Czoska A, Biernacka K, Balwicki (2021). Vaccine misinformation on social media - topic-based content and sentiment analysis of Polish vaccine-deniers' comments on Facebook. Hum Vaccin Immunother.

[60] Kata A (2012). Anti-vaccine activists, Web 2.0, and the postmodern paradigm--an overview of tactics and tropes used online by the anti-vaccination movement. Vaccine.

[61] Cianciara D, Szmigiel A (2019). Posting on „Nie szczepimy („We don’t vaccinate”) internet forum. Przegl Epidemiol.

[62] Griffith J, Marani H, Monkman H (2021). COVID-19 vaccine hesitancy in Canada: Content analysis of tweets using the Theoretical Domains Framework. J Med Internet Res.

[63] Thelwall M, Kousha K, Thelwall S (2021). Covid-19 vaccine hesitancy on English-language Twitter. EPI.

[64] Rovetta A (2021). Reliability of Google Trends: Analysis of the limits and potential of web infoveillance during COVID-19 pandemic and for future research. Front Res Metr Anal.

[65] Montalti M, Soldà G, Di Valerio Z, Salussolia A, Lenzi J, Forcellini M, Barvas E, Guttmann S, Messina R, Poluzzi E, Raschi E, Riccardi R, Fantini MP, La Fauci G, Gori D, San Marino Republic COVID ROCCA Group (2021). ROCCA observational study: Early results on safety of Sputnik V vaccine (Gam-COVID-Vac) in the Republic of San Marino using active surveillance. EClinicalMedicine.

[66] Gee J, Marquez P, Su J, Calvert GM, Liu R, Myers T, Nair N, Martin S, Clark T, Markowitz L, Lindsey N, Zhang B, Licata C, Jazwa A, Sotir M, Shimabukuro T (2021). First month of COVID-19 vaccine safety monitoring - United States, December 14, 2020-January 13, 2021. MMWR Morb Mortal Wkly Rep.

[67] Pagotto V, Ferloni A, Mercedes Soriano M, Díaz M, Braguinsky Golde N, González MI, Asprea V, Staneloni MI, Zingoni P, Vidal G, Aliperti V, Michelángelo H, Figar S (2021). Active monitoring of early safety of Sputnik V vaccine in Buenos Aires, Argentina. Medicina (B Aires).

[68] Elnegaard S, Andersen RS, Pedersen AF, Larsen PV, Søndergaard J, Rasmussen S, Balasubramaniam K, Svendsen RP, Vedsted P, Jarbøl DE (2015). Self-reported symptoms and healthcare seeking in the general population--exploring "The Symptom Iceberg". BMC Public Health.

[69] Sæbø O, Federici T, Braccini AM (2020). Combining social media affordances for organising collective action. Information Systems Journal.

[70] Kowalski P (2011). About the inevitabl excrement and defecation (in Polish). Colloquia Anthropologica et Communicativa: Ciało cielesne.

[71] Epstein S (1996). Impure science: AIDS, activism, and the politics of knowledge. Med Soc (Berkeley).

[72] Shimabukuro TT, Kim SY, Myers TR, Moro PL, Oduyebo T, Panagiotakopoulos L, Marquez PL, Olson CK, Liu R, Chang KT, Ellington SR, Burkel VK, Smoots AN, Green CJ, Licata C, Zhang BC, Alimchandani M, Mba-Jonas A, Martin SW, Gee JM, Meaney-Delman DM, CDC v-safe COVID-19 Pregnancy Registry Team (2021). Preliminary findings of mRNA Covid-19 vaccine safety in pregnant persons. N Engl J Med.

[73] Rogers EM (2010). Diffusion of innovations.

[74] Gopalsamy R, Semenov A, Pasiliao E, McIntosh S, Nikolaev A (2017). Engagement as a driver of growth of online health forums: Observational study. J Med Internet Res.

